# The Implications of Taste and Olfaction in Nutrition and Health

**DOI:** 10.3390/nu15153412

**Published:** 2023-07-31

**Authors:** Melania Melis, Iole Tomassini Barbarossa, Giorgia Sollai

**Affiliations:** Department of Biomedical Sciences, University of Cagliari, 09042 Monserrato, CA, Italy

Taste and olfaction are sensory modalities that act synergistically to orchestrate the behaviors essential for survival, such as interactions with the environment, nutrient-rich food identification, and the avoidance of noxious substances. Olfaction participates in long-range recognition, while taste mediates short-range detection. A critical investigation into how chemicals are detected at the periphery and how this information is conveyed and integrated at the central level could shed additional light on these processes, such as food intake regulation, eating behavior, and physio-pathological mechanisms. This Special Issue of Nutrients, “*Implications of Taste and Olfaction in Nutrition and Health*”, includes two reviews and eighteen original articles, which highlight the specific roles played by the gustatory and olfactory systems in influencing food preferences, diet, nutrition, and health. In particular, it has been highlighted how the chemosensory perception and the factors that modify and/or condition it can impact the health of individuals in different physiological states, such as age and sex. Furthermore, new techniques for the analysis and classification of individuals have been proposed in relation to their chemosensory function.

Taste and smell, precisely because of their function of perceiving chemical molecules, are mainly responsible for the choices of food and eating behavior of individuals, particularly by influencing their body weight and state of health [1,2,3,4,5,6,7,8,9,10,11]. Interestingly, several papers published in this Special Issue have brought about new insights into the relationship between chemosensory dysfunction, eating preferences, and mental and physical well-being. A growing body of literature suggests that olfactory and gustatory dysfunctions are early indicators of both frailty and mortality [12,13,14]. In this respect, the study by Vohra et al. [15] evaluated the association between dietary patterns and frailty status in older adults with olfactory dysfunction, showing that dietary patterns high in protein/selenium and b-carotene/vitamin A were associated with a lower prevalence of frailty in adults with olfactory dysfunction. Instead, no dietary pattern was associated with any measure of frailty in normosmic people. Although the relationship between olfactory dysfunction and frailty is multifaceted, the results of this study suggested that dietary changes may be a potential pathway underlying this relationship. Rawal et al. [16] found that adults aged 40 years and older with olfactory dysfunction ate foods with a higher energy density and, consequently, their diet was characterized by a higher consumption of sugars and saturated fats and a reduced consumption of vegetables. Moreover, differences related to age and gender were highlighted, showing that olfactory dysfunction may have a more negative impact on diet in middle-aged women, who prefer saturated fats, compared to similarly aged men or older men and women. Subsequently, Bhutani and McClain [17] showed that olfactory dysfunction can diminish flavor perception and reduce enjoyment in eating and drinking. This determines a shift in eating habits towards an unhealthy Western-style diet rich in calories aimed at compensating for this lack of pleasantness. The study by Brondel et al. [18] hypothesized that obesity results from an increased brain activation of the reward circuitry during the processing of food flavors and aromas, most likely due to the reinforcing value of palatable foods high in fat and carbohydrates. This hypothesis would explain both the high consumption of fatty foods and therefore the decrease in the gustatory sensitivity to fats by negative feedback mechanisms. Preferences for unhealthy foods such as fats and sugars appear to be related to a taste sensitivity to carbohydrates. In fact, Costanzo et al. [19] showed that individuals with a greater sensitivity to carbohydrates increase not only the intake of these, but also of foods with a high energy content, with a consequent increase in body mass. The study by Ervina et al. [20] aimed to investigate the relationship between gustatory sensitivities and pleasantness in preadolescents, with the scope of elucidating whether the pleasant sensation of sweetness can be used as a strategy to suppress the unpleasant ones of bitterness and acidity, which often contribute to the refusal of foods such as fruit and vegetables by preadolescent boys. Their results showed that the sweetness-suppressing effect on bitterness and acidity sensations was associated with individual taste sensitivity. Children who were highly sensitive to bitterness and acidity and less sensitive to sweetness did not experience a suppression effect of sweetness on these sensations. Conversely, children less sensitive to bitter and sour flavors showed a greater liking as the sucrose concentrations were increased. This result proves that specific strategies need to be developed for children’s taste response profiles to increase their acceptance of foods dominated by warning sensations of acidity and bitterness, such as fruits and vegetables. The use of the gustatory function as a strategy to modify and/or condition the eating habits of adolescents was also investigated in the study by Proserpio et al. [21]. Indeed, the period of adolescence is often characterized by a considerable risk of weight gain due to the high consumption of foods rich in sugars. The results obtained showed that the adolescents examined had both a different gustatory function and different multisensory process involved in the perception of food in relation to their nutritional status. The overweight subjects had a weaker ability to identify the gustatory function, while they seemed to pay more attention to the odors/stimuli that signaled high-calorie products. If the way the chemical senses perceive sensory information could be changed, innovative food formulations could be developed with, for example, a reduced amount of sugar, leading to a potential decrease in caloric intake, helping to tackle the obesity epidemic.

A particularly interesting aspect to consider is that there is a large inter-individual variability in gustatory and olfactory perception, especially when considering the use of chemosensory function as a strategy to combat obesity and/or unhealthy eating behaviors. The factors traditionally considered to be responsible are physiological, genetic, environmental, and cultural [22,23,24,25,26,27,28,29]. Two studies proposed by Melis et al. highlighted the role of genetic variability in both olfactory [30] and gustatory [31] perception. The first study highlighted the role of the olfactory system and its individual variability in the regulation of body weight, not only by acting on the quality and quantity of the food that is ingested, but also on the energy and basal metabolism, through the activity of the voltage-gated potassium Kv1.3 channels. In particular, the study showed that olfactory function and body weight are affected by the *rs2821557* (T/C) polymorphism of the human Kv1.3 gene: subjects who were TT homozygous or heterozygous exhibited lower BMIs and achieved higher olfactory scores than those with the CC genotype. The results were sex-dependent: heterozygous females performed better than heterozygous males, suggesting an involvement of this polymorphism of the human Kv1.3 gene in the physiological variability of olfactory function linked to sex. The results also highlighted that a normosmia condition appears to balance the BMI differences shown between heterozygous males and females. Individual differences in sweet taste sensitivity, influenced by genetic and environmental variables, can affect dietary preferences and nutritional status. In the second study, Melis et al. [31] determined the effect of the well-established factors influencing general taste variability, such as sex and the density of fungiform papillae, SNP-specific genetic variants of TAS1R2 receptor genes, and TAS1R3 and non-specific genetic factors (PROP phenotype and genotype), on threshold and suprathreshold sweet taste sensitivity. This represents the first analysis of the different genetic and non-genetic factors together that are involved in sweet taste sensitivity. The findings confirmed the role of the PROP phenotype in the taste perception of sweets and showed a higher sweet sensitivity in females with a lower BMI compared to males. Additionally, the findings showed, for the first time, the involvement of the *rs35874116* SNP of TAS1R2 in the sweet taste sensitivity to low concentrations of sweets in normal-weight subjects. The results showed that males, PROP non-tasters, and subjects with the TT genotype of the *rs35874116* SNP of TAS1R2, with a low sensitivity for sweets, could be more vulnerable to a high sugar consumption and thus have a greater risk of an increased BMI or metabolic disease.

Repeated exposure to particular substances and/or habits with certain foods can influence eating behavior by changing food preferences and therefore body weight. The review by Wilk et al. [32] emphasized that an excessive consumption of foods high in sugar is currently one of the most important factors leading to the development of the global obesity pandemic. Additionally, there is evidence that obesity contributes to a reduced sensitivity to sweet tastes and hormonal changes that affect the appetite, leading to an increase in the desire for sweets. A high intake of sugar increases the caloric value of the diet and, consequently, leads to weight gain. A potential method for reducing the energy value of diets while maintaining sweet tastes is the use of non-nutritive sweeteners (such as aspartame, acesulfame-K, sucralose, saccharin, and cyclamate, etc.), commonly used as table sugar substitutes, as they have a high sweetness with almost no calories due to their high sweetening power. Since food preferences and eating habits are set from a young age, a food education intervention is needed to reduce the consumption of sugar-rich products. In this regard, the study by Agarwal et al. [33] showed that prenatal exposure to caffeine of mothers was positively associated with a high body mass index (BMI) in children. Why this association occurred was unclear, but it appears that prenatal caffeine exposure impairs the *in utero* development of the brain structures associated with food preferences (such as the frontal lobe), leading to a higher total sugar intake later in life in childhood. The authors concluded that caffeine consumption should be reduced during pregnancy and that it would be advisable to start an awareness program to avoid these negative consequences on the child’s development. The association between taste sensitivity and alterations in brain structures and functions was also studied by Wei et al. [34]. These authors analyzed the genetic association between the perception of sweet or bitter beverages and human brain proteins, since the perception of a bitter or sweet drink is associated with alterations in brain structure and function [35,36]. Their results supported the potential effect of bitter or sweet drink perception on brain function and identified several candidate brain proteins for bitter or sweet drink perception. Diet and salivary proteins influence the composition of the oral microbiome, and recent data have suggested that TAS2R38 bitter taste genetics may also play a role. Accordingly, the study by Yousaf et al. [37] investigated the effects of daily exposure to a cranberry polyphenol oral rinse on taste perception, salivary proteins, and oral microbiota. Correlation networks between the oral microbiota, salivary proteins, and sensory evaluations showed that the PROP super tasters’ (STs) microbiome had a more complex relationship with the salivary proteins, especially proline-rich proteins, than that of the PROP non tasters (NTs). These results showed that the cranberry polyphenol extract modulated the oral microbiome of the NTs to be similar to that of the STs, which may have implications for oral health. This first investigation on the interconnection capabilities of taste, salivary proteins, and the oral microbiome in PROP-classified individuals represents a path forward for taste researchers interested in studying the highly intertwined nature of the genetics of taste, eating behavior, and oral health.

The inter-individual variability of taste sensitivities associated with genetic factors was also studied by Cecati et al. [38]. These authors verified whether the presence of four different single-nucleotide polymorphisms (SNPs) in the genes coding for the bitter (TAS2R38; 145G > C; 785T > C) and sweet (TAS1R3; I1572C > T; I1266C > T) taste receptors may influence the recognition of basic tastes, and whether the allelic distribution of these SNPs varied according to BMI. Their results showed that the interindividual ability to identify sweet tastes at different concentrations was related to the presence of at least one genetic variant for the bitter receptor gene, but not to BMI, suggesting that research is still far from completely identifying the causes of complex pathologies such as obesity and that additional knowledge still needs to be acquired in the field of food preferences, choices, and intake in relation to genetic factors, including the taste receptors of the gut. Two studies on mammals underlined the role of genetic factors in the sensory perception of fats. Schumann et al. [39] showed that a progranulin deficiency leads to a decreased expression of CD36 in the tongue, resulting in a greater preference for greasy tastes and fatty foods. When applied to humans, these data suggest that patients with diseases associated with low progranulin levels (for example, compulsive eating associated with frontotemporal dementia, psychiatric diseases, and comorbid obesity) may have a high preference for fatty tastes. Ullah et al. [40] showed that mRNA coding for leptin (an anorectic hormone that regulates food intake, energy expenditure, and body weight) and the leptin receptor (Ob-Rb) was expressed in the taste bud cells of mice (TBC). The results suggested that leptin in the tongue exerts an inhibitory action on fatty acid preference by interfering with the Ca^2+^ signal (it reduces the intracellular Ca^2+^ levels) and the membrane potential (it causes hyperpolarization in TB). This study contributes to the hypothesis that leptin may regulate the intake of high-fat foods through its action in the periphery by influencing the early mechanisms of fat taste perception in the microenvironment of the taste buds.

An interesting aspect concerns the association between subjective-physiological emotional coherence and greater well-being, as well as between subjective-physiological emotional coherence and superior nutritional status, in older populations. Accordingly, the study by Saito et al. [41] compared the subjective-physiological emotional coherence during food consumption between older and younger adults, by the means of facial electromyography. The results revealed that the emotional coherence between subjective experiences and the facial electromyography (EMG) activity during food consumption was maintained or increased in older adults as compared to younger ones, suggesting that the essential function of emotions is maintained in older adults, which probably enhances the quality of health and life in late adulthood. EMG was also used by Sato et al. [42] to evaluate the subjective hedonic and emotional experiences during eating. Their results demonstrated that the subjective ratings of liking, wanting, and valence were negatively associated with corrugator supercilii electromyography and positively associated with masseter and suprahyoid electromyography during food consumption with mastication, suggesting that subjective hedonic experiences during the consumption of food can be sensed using EMG signals from the brow and masticatory muscles.

The taste and smell function in healthy young and middle-aged females and males were studied by Lim et al. [43]: they examined the degree to which taste and smell sensitivity are related to taste and smell preferences, respectively, the extent to which the thresholds for different tastes align with each other, and the association between taste and smell sensitivity. The results showed that: (1) the taste thresholds were highly correlated, but no correlation was observed between the taste and smell thresholds and between the thresholds and preference, (2) women were more sensitive to both taste and smell than men, and (3) no effect of age on sensitivity and no effect of sensitivity on quality of life were found, collectively indicating the independence of taste and smell, despite their overlap during sensory experiences.

Recent studies have shown that the latest tools in bioinformatics can be used to classify subjects to study the correlations among variables [28,44]. Accordingly, the study by Naciri et al. [45] evaluated the possible use of novel bioinformatics techniques, such as the Supervising Learning approach (SL), for the automatic identification of PROP taster categories (super taster (ST); medium taster (MT); and non-taster (NT)). The results showed that SL, exploiting the biological characteristics of the subjects, allowed for the objective, automatic, and immediate identification of the PROP taster status, with an accuracy of 97%. Among the biological characteristics of the subjects, the perceived intensity for the high concentrations of PROP and the density of the taste papillae were the most important, while the AVI/AVI, PAV/PAV, and PAV/AVI genotypes of *TAS2R38* were significant for classifying NT, ST, and MT, respectively. These results suggested that the SL approach can represent an objective and reliable tool in studies on the physiology of taste, with applications ranging from basic science and medicine to food science. Indeed, PROP taste sensitivity has been used extensively to assess inter-individual taste variability and its impact on food preferences, nutrition, and health.

In conclusion, the studies featured in this Special Issue, as well as in the wider literature cited in these articles and reviews, represent the state of the art of our knowledge on the relationship between chemosensory systems and the nutritional health statuses of individuals. As the biological, behavioral, and environmental mechanisms underlying feeding behavior continue to be investigated and the causes and consequences of changes in the gustatory and olfactory functions are better characterized, it is possible to implement, study, and evaluate new intervention strategies. These results will not only help to better understand the relationship between chemosensory perception and its individual variability and eating behavior, but they can also help healthcare professionals to educate their patients and facilitate the adoption of healthy eating behaviors.
